# Implementation studio: implementation support program to build the capacity of rural community health educators serving immigrant communities to implement evidence-based cancer prevention and control interventions

**DOI:** 10.1007/s10552-023-01743-6

**Published:** 2023-07-13

**Authors:** Linda K. Ko, Thuy Vu, Sonia Bishop, Jennifer Leeman, Cam Escoffery, Rachel L. Winer, Miriana C. Duran, Manal Masud, Yaniv Rait

**Affiliations:** 1 Department of Health Systems and Population Health, Health Promotion Research Center, University of Washington, Seattle, WA USA; 2 Division of Public Health Sciences, Fred Hutchinson Cancer Center, Seattle, WA USA; 3 School of Nursing, The University of North Carolina at Chapel Hill, Chapel Hill, NC USA; 4 Department of Behavioral, Social and Health Education Sciences, Rollins School of Public Health, Emory University, Atlanta, GA USA; 5 Department of Epidemiology, University of Washington, Seattle, WA USA; 6 Department of Health Systems and Population Health, Hans Rosling Center for Population Health, University of Washington, 3980 15Th Avenue NE, 4Th Floor, UW Mailbox 351621, Seattle, WA 98195 USA

**Keywords:** Implementation plan, Evidence-based intervention, Implementation studio, Community-based organizations, Community health educators, Rural setting

## Abstract

**Purpose:**

Rural community-based organizations (CBOs) serving immigrant communities are critical settings for implementing evidence-based interventions (EBIs). The Implementation Studio is a training and consultation program focused on facilitating the selection, adaptation, and implementation of cancer prevention and control EBIs. This paper describes implementation and evaluation of the Implementation Studio on CBO’s capacity to implement EBIs and their clients’ knowledge of colorectal cancer (CRC) screening and intention to screen.

**Methods:**

Thirteen community health educators (CHEs) from two CBOs participated in the Implementation Studio. Both CBOs selected CRC EBIs during the Studio. The evaluation included two steps. The first step assessed the CHEs’ capacity to select, adapt, and implement an EBI. The second step assessed the effect of the CHEs-delivered EBIs on clients’ knowledge of CRC and intention to screen (*n* = 44).

**Results:**

All CHEs were Hispanic and women. Pre/post-evaluation of the Studio showed an increase on CHEs knowledge about EBIs (pre: 23% to post: 75%; *p* < 0.001). CHEs’ ability to select, adapt, and implement EBIs also increased, respectively: select EBI (pre: 21% to post: 92%; *p* < 0.001), adapt EBI (pre: 21% to post: 92%;* p* < 0.001), and implement EBI (pre: 29% to post: 75%; *p* = 0.003). Pre/post-evaluation of the CHE-delivered EBI showed an increase on CRC screening knowledge (*p* < 0.5) and intention to screen for CRC by their clients.

**Conclusion:**

Implementation Studio can address unique needs of low resource rural CBOs. An implementation support program with training and consultation has potential to build the capacity of rural CBOs serving immigrant communities to implementation of cancer prevention and control EBIs. Clinical Trials Registration Number: NCT04208724 registered.

## Background

Accelerating the implementation of evidence-based cancer prevention and control interventions in settings that reach rural communities is critical [1, 2]. Marked disparities in breast, cervical, and colorectal cancer incidence and mortality rates were reported among racial and ethnic minorities living in rural areas [3–5] due to higher poverty rates, lower levels of education, lack of health insurance, and fewer healthcare providers practicing in rural areas compared to urban areas [6, 7]. There is significant evidence that screening efforts can reduce cancer disparities [1–3]

Evidence-based interventions (EBIs) improve cancer screening rates, but implementation of EBIs is low in rural communities [1, 2]. Community-based organizations (CBOs) play an important role in cancer prevention and control [8]. In addition to promoting health education, CBOs are often connected with healthcare systems and can serve as a link between the community and the healthcare system by providing community members with screening referrals, home health education, and connections to health and social services [8, 9]. However, many CBOs show low awareness of EBIs and how to find, use, adapt, and implement them.

The Implementation Studio is a structured training and consultation program focused on facilitating the awareness, selection, adaptation, and implementation of cancer prevention and control EBIs, and building deliberate partnerships with rural CBOs. At the heart of the Implementation Studio is the implementation blueprint, which is created at the beginning of the Studio, and revisited throughout both the training and consultation period in an iterative process. The Implementation Studio deliberately uses the implementation blueprint by aligning blueprint components with the Studio’s capacity building activities; these include EBI selection and adaptation, identification of steps to carry out the implementation details in the blueprint, brokering stakeholders’ collaboration and partnerships, and monitoring performance.

Development of the Implementation Studio’s content was extensive and systematic to help ensure its accessibility to rural and limited-English-proficient communities, respectively. Broadly, the Studio was informed by both published literature focused on capacity building and the Putting Public Health Evidence in Action (PPHEIA) curriculum created by the Cancer Prevention and Control Research Network (located at https://cpcrn.org/training) [10–12], respectively. The PPHEIA curriculum is aimed at facilitating EBI implementation by CBOs [10, 11, 13]; it focuses on didactic learning to builds skills in how to find, select, and implement EBIs.

We adapted existing training content to be culturally and linguistically appropriate for Hispanic communities using the cultural and linguistic adaptation framework [14, 15]. Specifically, we appraised published trainings to identify core elements (training on EBIs and identifying CBO resources), structure (didactic), and theoretical underpinnings (increase knowledge and awareness) to build upon. We subsequently reviewed the literature on capacity building training for CBOs and critical elements to fill the gaps (skill building framework, use of implementation blueprint, partnership facilitation, EBI adaptation, and training and education). We then assessed the rural context and characteristics of rural CBOs, including availability of resources, organizational size, and partnerships. We solicited input from rural CBOs through key informant interviews to further inform and strengthen our efforts. The Implementation Studio was created upon synthesis and summary of information from these stepwise activities*.*

This paper describes implementation and evaluation of the Implementation Studio delivered to community health educators (CHEs) employed by rural CBOs who serve limited-English-proficient community. The evaluation assessed (1) the CBO CHEs’ awareness of EBIs and their capacity to select, adapt and implement a cancer screening EBI and (2) community members’ intention to screen for cancer after participating in the cancer screening EBI delivered by their CBO.

## Methods

### Overview

This evaluation study includes a description of the Implementation Studio and the quantitative tools used to evaluate the Implementation Studio on the capacity of the CHEs as well as to assess colorectal cancer (CRC) screening knowledge and intention to screen by community members.

### Description of the implementation studio

The Implementation Studio curriculum includes five key steps: (1) creation of an implementation blueprint, (2) review of EBIs and adaptation to a rural context, (3) stakeholder collaboration and partnership building, (4) training and education, and (5) EBI implementation and monitoring. Two separate Studios were delivered to each CBO who participated with their team of CHEs. The Studios were delivered by the University of Washington [UW] and included bilingual and bicultural team members who were instrumental in the development of the program. The Implementation Studio includes 8 hours of content; it can be delivered as a two half-day workshop or eight, 1-h workshops. The format can be in-person or via videoconferencing, followed by biweekly consultations through phone, video conferencing, or in-person.

CHEs from one CBO chose two half-day workshops, while the second CBO chose eight 1-h training workshops. One studio was conducted in English as community health educators were bilingual (Spanish–English) and the second studio in Spanish to accommodate their Spanish language dominant CHEs. Delivery of the Spanish Studio was facilitated by a bicultural and bilingual research team member using materials translated in the Spanish language.

The first half of the studio covered steps 1–3 described above under ‘Description of the Implementation Studio.’ The second half of the studio occurred one to 2 weeks after the first half of the studio based on CHEs’ readiness to revise their blueprints. The focus of the latter half of the Studio covered steps 4–5, to identify emergent knowledge gaps among CBO staff, deliver additional training to address the gaps, and further fine-tune the blueprints. The training was recorded; recordings and presentation slides were uploaded to a shared folder for the CHEs to access for self-directed learning or review in the event of staff turnover or the need for refresher training. The CHEs were ready to begin implementation of the EBI they selected within 2 weeks of the second half of the studio. We will illustrate the content and the delivery of the Implementation Studio using two CBOs who completed the training, selected and implemented CRC screening EBIs, and who participated in evaluation of the Implementation Studio.

*Step 1. implementation blueprint* Prior to the first studio session, each CBO team drafted their initial implementation blueprint using a template provided by the UW support team. Powell et al. define implementation blueprint as a plan that includes goals, strategies, the scope of change, timeframe and milestones, and progress measures [16]. The blueprint template included fields to specify implementation duration (either 6 months or 12 months based on their organizational capacity), and their goals and strategies. One CBO selected a 6-month blueprint, while the second CBO selected a 12-month blueprint. The blueprint template also captured the type of cancer they would like to address (e.g., breast, cervical, or colorectal cancer). Both CBOs independently chose to focus on CRC screening given the high prevalence, incidence, and mortality among Hispanics after viewing the American Cancer Society Facts and Figures.

CBO teams sent their draft implementation blueprints back to the UW support team, leaving blank the sections they needed UW support to complete. The development of the blueprint was iterative with revisions made as needed throughout the Studio process. The UW support team guided CBOs on their creation of a structured implementation blueprint based on the cancer topic they selected. The blueprint included (1) aim/purpose of implementing the cancer-specific screening EBI, (2) scope of the change (e.g., what organizational unit will take on the EBI), (3) action steps, timeframe, and milestones, (4) appropriate performance measures to track EBI activities, (5) training needs to carry out the work, and (6) plans for execution.

*Step 2. Review of EBIs and adaptation to rural context* In this step, the UW support team reviewed the blueprint with the CHEs and created a menu of EBIs that matched the (1) CBO’s goals and strategies, (2) resources available for the CBO to implement an EBI, and (3) the feasibility of accomplishing the task within the 6- or 12-month timeframe the CBO specified. The UW support team guided the CHEs on where to find EBIs: guidance included providing the CHEs with activities and exercises to practice navigating online EBI resources to find and select the EBI most appropriate for their CBO and community needs. Together, the UW support team and CHEs reviewed the Community Guide [17] and the NCI’s Evidence-Based Cancer Control Programs (EBCCP). For the Spanish language Studio, a bilingual/bicultural research team member provided real-time translation while navigating the website which is only available in English. Both CBOs selected a practitioner-delivered CRC education strategy (ACCION) from the EBCCP website [18].

Once the EBI was selected, the Studio session focused on adaptation. The UW support team trained the CHEs on how to adapt the EBI to their setting using the four-step cultural and linguistic adaptation process developed by the UW support team in collaboration with the University of North Carolina team [14]. The adaptation process included (1) appraisal of the EBI, (2) review of the literature, (3) assessment of the regional context, and (4) soliciting direct input from the CBO staff and clients. CBO-led adaptations to the CRC EBI included incorporation of images that more closely resemble their community members, rewording text to enhance use of lay language, and editing screening guidelines to reflect recently revised recommendations to begin average risk CRC screening at age 45. All adaptations occurred at pre-implementation were proactive, fidelity consistent, and made to accommodate the rural context [19].

*Step 3. Stakeholder collaboration and partnership building* In this step, CHEs listed resources (e.g., funding, staff time, specific skills) that they currently have and created a list of other resources and new partnerships (e.g., with healthcare systems) that they need to reach the goals set in their blueprint. The CHEs were asked to indicate clear steps describing how their partnerships could support them to accomplish the plan in their blueprint. CBOs were not able to execute this step as the studio took place in the first year of the COVID-19 pandemic (during November 2020 to December 2020), when it was not feasible to engage healthcare partners who were already overwhelmed responding to the pandemic. Health system partners would have provided navigation to help community members complete their CRC screening and obtain follow-up services (such as diagnostic or treatment), if needed. In the absence of these partnerships, the UW support team connected the CBOs with the Washington State Department of Health, an awardee of the CDC Colorectal Cancer Screening Program, to access program resources and regional CRC screening contacts who might be able to provide or advise on potential follow-up services.

*Step 4. Training and education* the UW support team asked CHEs to provide information on (1) staff training and education needs, (2) staff members who need the training, (3) materials and experts who can provide the training, and (4) access to the training materials and experts. All CHEs received training on how to culturally and linguistically adapt evidence-based interventions to their setting and in program evaluation.

The UW support team provided additional training that were not accessible to the CHEs through their organizations. During the studio, the UW support team asked clarifying questions to help identify training materials, in the form of prints, self-directed learning videos, or in-person/virtual training delivered by a content expert. Topics covered included education on United States Preventive Services Taskforce guidelines for cancer screening, qualitative data collection methods with focus groups and semi-structured interviews, best practices for conducting survey data collection, and analyzing and interpreting data.

*Step 5. Implementation steps and performance monitoring* Finally, the session reviewed the process of rolling out the activities step by step, how to track EBI activities, and how to assess the costs of roll out. The UW support team provided the CHEs with an implementation tracking sheet to monitor the implementation steps (e.g., implementation activities, date of implementation, person assigned to complete the activity), human (e.g., labor costs), and capital (non-labor costs) resources required to complete those steps [20, 21]. The Studio also included activities that were completed during and outside of the studio.

### Implementation of the implementation blueprint

#### Regular consultations

Once implementation of the EBIs were underway at the CBO, the UW support team provided regular consultations with each CBO team. During these consultations, the research team and the CHEs reviewed progress, discussed successes, and addressed unforeseen barriers to implementation. The consultations were delivered remotely via a conference call or virtual meeting platform and each consultation lasted approximately 30 min. The consultations served as opportunities for the research team to provide technical assistance, data review, and support during implementation.

### Evaluation of the studio and implementation impacts

The evaluation involved two-tiered steps and was conducted by the research team. First, in tier one we evaluated the impact of the Implementation Studio on each CBO team member’s knowledge, ability and self-efficacy to select, use and implement EBIs using a pre/post-survey [10]. After the CBO delivered the EBI workshop that they selected and adapted, we then conducted tier two of our evaluation which focused on the CBO clients who participated in the EBI workshop. We use the term, “client” to include both individuals who may have received or accessed services offered by the CBO, in addition to their community members at large. The client-focused evaluation assessed clients’ knowledge of CRC and CRC screening, as well as intention to get screened for CRC through a self-reported pre/post-survey.

#### CBO recruitment

We sent two email messages with recruitment flyers to CBOs with whom we collaborated in previous studies. When CBOs responded to the email request, a research team member provided more information about the study, assessed eligibility (CHEs serving rural communities), described what participation in the Implementation Studio would entail, and answered questions from the CBOs. The CBOs signed a consent form and received $5,000 to offset the costs of implementing the EBIs. We recruited two CBOs and 13 CHEs employed by the CBOs. Both CBOs’ services were mainly directed to the Hispanic community. The first CBO was located in rural Yakima Valley of the Eastern Washington State. The second CBO was located in Seattle, but employed CHEs who were residing and working in rural farming regions of Washington State, including Mount Vernon and Yakima Valley. Both CBOs provide social services to limited-English-proficient community members in their respective regions; the social services they provide include education programming (e.g., ESL, citizenship/naturalization classes, oral health, mental health) and language translation services.

#### Client recruitment

Each participating CBO was asked to recruit 25 clients from their community network to receive the CRC screening EBI workshop they adapted. The CBOs promoted the study to their clients, who were participating in their existing programming or in the community using promotional flyers created in Spanish. When community clients contacted the CBOs with interest in participating in the study, the CBOs gathered their name and contact information. The research team then contacted the clients by telephone to assess eligibility, obtain verbal consent for participation in the study, and administer the pre- and post-surveys.

#### CBO data collection

Data collection was conducted by a bilingual/bicultural research team member who was not involved in the delivery of the Implementation Studio. Pre/post-surveys with CBOs were adapted from Escoffery and colleagues from Emory University [10] and assessed (1) knowledge of implementation blueprints and EBIs, (2) ability to develop and use an implementation blueprint, ability to develop, use, select, adapt and implement EBIs, and (3) self-efficacy (measured as confidence) to develop and use a blueprint, and self-efficacy to use, select, adapt, and implement EBIs. These questions had five response categories (low, moderately low, moderate, moderately high, and high). For analysis, moderately low was collapsed with low and moderately high was collapsed with high.

Pre/post-Client Surveys were conducted with those who participated in the CRC screening EBI workshop that was delivered by CBO CHEs. The surveys included statements that assessed clients’ knowledge about topics covered in the EBI workshop, including CRC facts, CRC screening guidelines, and benefits of CRC screening. The CRC questions were adapted from the National Health Interview survey [22] and studies on CRC screening studies among Latinos [23, 24]. These statements had three response categories (agree, disagree, and I am not sure). The post-survey also included questions that measured the extent clients learned about CRC and CRC screening using four response categories. For example, “As a result of participating in this workshop, my knowledge about CRC, stayed the same, increased a little, increased somewhat, and increased a lot.” The four response categories were transformed into numeric values for analysis (1 = stayed the same to 4 = increased a lot). We also assessed clients’ likelihood of getting screened for CRC, talking to a doctor about getting screened for CRC, and talking to a family member or friends about the importance of screening for CRC. Response options consisted of four categories ranging from “not likely” to “very likely.” For analysis, the responses were transformed into numeric values (1-not likely to 4-very likely). The post-survey also included questions that assessed clients’ overall perception of the EBI workshop including overall content, presenter, and amount of information. These questions had four response categories (strongly agree, somewhat agree, somewhat disagree, and strongly disagree). For analysis, these data were transformed into numeric values (1 = strongly agree to 4 = strongly disagree).

#### Analysis

Data were captured using research electronic data capture (REDCap). We examined data separately by CBO as well as cumulatively, with combined CBO data. Descriptive analyses were generated with frequencies for categorical variables and means and standard deviation for continuous variables. Bivariate analyses were conducted with Fisher’s exact test of significance for categorical variables and paired *t* tests for continuous variables.

## Results

### Demographic characteristics of CBO CHEs

Table 1 shows the demographics of the CHEs employed by the CBOs who participated in the Implementation Studio (*n* = 14); 14 CHEs participated in the Implementation Studio, but only 13 CHEs provided their demographic information. All participants were Hispanic and women. The CHEs were equally distributed in language(s) spoken reporting bilingual Spanish dominant (29%), bilingual English dominant (29%), and Spanish only (29%). No one reported speaking English only. The majority of the CBO staff were born outside of the USA (86%) and were uninsured (57%). About half of the CBO’s CHEs were employed part-time (43%) and had a high school degree (50%); one participant (7%) had an advanced degree.

**Table 1:** Demographic characteristics of CBO staff

	Total (*n* = 14)		CBO 1		CBO 2	
Mean age (SD)*	40.1	(9.71)	41	(7.79)	39.8	(10.2)
Ethnicity (n, %)						
Hispanic	13	93%	3	100%	10	91%
Non-Hispanic	–	–	–	–	–	–
Prefer to not answer	1	7%	–	–	1	9%
Language (n, %)						
Bilingual (Spanish Dominant)	4	29%	–	–	4	36%
Bilingual (English Dominant)	4	29%	3	100%	1	9%
Spanish Only	4	29%	–	–	4	36%
English Only	–	–	–	–	–	–
Prefer to not answer	2	14%			2	18%
Gender (n, %)						
Man	–	–	–	–	–	–
Woman	13	93%	3	100%	10	91%
Prefer to not answer	1	7%	–	–	1	9%
Employment Status (n, %)						
Full-Time	6	43%	3	100%	3	27%
Part-Time	7	50%	–	–	7	64%
Prefer to not answer	1	7%			1	9%
Marital Status (n, %)						
Married or living with a partner	8	57%	1	33%	7	64%
Single	5	36%	2	66%	3	27%
Prefer to not answer	1	7%	–	–	1	9%
Country of Birth (n, %)						
USA	1	7%	1	33%	–	–
Outside the USA	12	86%	2	67%	10	91%
Prefer to not answer	1	7%	–	–	1	9%
Mean (SD) Year living in the USA*	15.75	(9.27)	25.5	(5.5)	13.8	(8.61)
Health insurance (n, %)						
Insured	5	36%	2	67%	3	27%
Uninsured	8	57%	1	33%	7	64%
Prefer to not answer	1	7%	–	–	1	9%
Education (n, %)						
Elementary school	1	7%	–	–	1	9%
High school graduation or GED	7	50%	–	–	7	64%
Some college (e.g., associate’s degree)	2	14%	2	67%	–	–
College graduate (e.g., BA/BS)	2	14%	1	33%	1	9%
Graduate school degree	1	7%	–	–	1	9%
Prefer to not answer	1	7%	–	–	1	9%
Annual household income (n, %)						
Less than $15,000	1	7%	–	–	1	9%
$15,000 to less than $35,000	4	29%	–	–	4	36%
$35,000 to less than $50,000	2	14%	1	33%	1	9%
$50,000 to less than $75,000	1	7%	1	33%	–	–
$75,000 to less than $100,000	2	14%	1	33%	1	9%
Don’t know	1	7%	–	–	1	9%
Prefer not to answer	3	21%	–	–	3	27%

* = Mean statistics do not include 1 participant who declined to answer all demographic questions

### Change in knowledge, ability, and self-efficacy among CBO CHEs

Table 2 shows significant change in the CHEs’ knowledge, ability, and self-efficacy to develop and use the implementation blueprint and EBIs pre/post-participation in the Implementation Studio. Specifically, CHEs were more likely to report high/moderately high knowledge about the blueprint (pre: 29% vs. post: 75%; *p* = 0.001) and EBIs (pre: 23% vs. post: 75%; *p* < 0.001) after participating in the Studio. Ability to develop (pre: 36% vs post: 75%; *p* = 0.005) and use the implementation blueprint (pre: 29% vs post: 83%; *p* < 0.001) also increased after participation in the Studio. The CHEs also reported greater ability to use EBIs (pre: 36% vs. post: 75%; *p* = 0.047), select EBIs (pre: 21% vs. post: 92%; *p* < 0.001), adapt EBIs (pre: 21% vs. post: 92%; *p* < 0.001), and implement EBIs (pre: 29% vs. post 75%; *p* = 0.003). CHEs also reported greater self-efficacy (measured as confidence) to develop (pre: 29% vs. post 83%; *p* < 0.001) and use an implementation blueprint (pre: 36% vs. post 83%; *p* < 0.006). There was also an increase in self-efficacy to use EBIs (pre: 36% vs post: 92%; *p* = 0.001), select EBIs (pre: 29% vs. post: 100%; *p* < 0.001), adapt EBIs (pre: 14% vs. post: 100%; *p* < 0.001), and implement EBIs (pre: 29% vs. 83%; *p* < 0.001).

**Table 2:** Pre/Post-evaluation of the implementation studio

	Total (*n* = 14)				*P* value	CBO1 (*n* = 3)				CBO2 (*n* = 11)			
	Pre		Post			Pre		Post		Pre		Post *	
Knowledge (n, %)													
IB					0.001								
Low/Moderately Low	7	50%	0	0%		2	67%	0	0%	5	45%	0	0%
Moderate	3	21%	3	25%		0	0%	0	0%	3	27%	3	33%
High/Moderately high	4	29%	9	75%		1	33%	3	100%	3	27%	6	67%
EBI**					< 0.001								
Low/Moderately Low	9	69%	0	0%		2	67%	0	0%	7	70%	0	0%
Moderate	1	8%	3	25%		0	0%	0	0%	1	10%	3	33%
High/Moderately high	3	23%	9	75%		1	33%	3	100%	2	20%	6	67%
Ability (n, %)													
Develop IB					0.005								
Low/Moderately low	7	50%	0	0%		2	33%	0	0%	5	45%	0	0%
Moderate	2	14%	3	25%		0	0%	0	0%	2	18%	3	33%
High/Moderately high	5	36%	9	75%		1	33%	3	100%	4	36%	6	67%
*Use IB*					< 0.001								
Low/Moderately low	7	50%	0	0%		2	67%	0	0%	5	45%	0	0%
Moderate	3	21%	2	17%		0	0%	0	0%	3	27%	2	22%
High/Moderately high	4	29%	10	83%		1	33%	3	100%	4	36%	7	78%
*Use EBI*					0.047								
Low/Moderately low	6	43%	0	0%		1	0%	0	0%	5	45%	0	0%
Moderate	3	21%	3	25%		1	33%	1	33%	2	18%	2	22%
High/Moderately high	5	36%	9	75%		1	33%	2	67%	4	36%	7	78%
*Select EBI*					< 0.001								
Low/Moderately low	7	50%	0	0%		1	33%	0	0%	6	55%	0	0%
Moderate	4	29%	1	8%		1	33%	0	0%	3	27%	1	11%
High/Moderately high	3	21%	11	92%		1	33%	1	100%	2	18%	8	89%
*Adapt EBI*					< 0.001								
Low/Moderately low	7	50%	0	0%		2	33%	0	0%	5	45%	0	0%
Moderate	4	29%	1	8%		0	0%	0	0%	4	36%	1	11%
High/Moderately high	3	21%	11	92%		1	33%	3	100%	2	18%	8	89%
*Implement EBI*					0.003								
Low/Moderately low	6	43%	1	8%		2	33%	0	0%	4	36%	1	11%
Moderate	4	29%	2	17%		0	0%	1	33%	4	36%	1	11%
High/Moderately high	4	29%	9	75%		1	33%	2	67%	3	27%	7	78%
Confidence (n, %)													
*Develop IB*					< 0.001								
Low/Moderately low	6	43%	0	0%		2	33%	0	0%	4	40%	0	0%
Moderate	3	21%	2	17%		0	0%	1	33%	3	30%	1	11%
High/Moderately high	4	29%	10	83%		1	33%	2	67%	3	30%	8	89%
*Use IB*					0.006								
Low/Moderately low	5	36%	0	0%		2	33%	0	0%	3	30%	0	0%
Moderate	3	21%	2	17%		0	0%	1	33%	3	30%	1	11%
High/Moderately high	5	36%	10	83%		1	33%	2	67%	4	40%	8	89%
*Use EBI*					0.001								
Low/Moderately low	5	36%	0	0%		1	33%	0	0%	4	40%	0	0%
Moderate	3	21%	1	8%		1	33%	1	33%	2	20%	0	0%
High/Moderately high	5	36%	11	92%		1	33%	2	67%	4	40%	9	100%
*Select EBI*					< 0.001								
Low/Moderately low	7	50%	0	0%		2	33%	0	0%	5	45%	0	0%
Moderate	3	21%	0	0%		0	0%	0	0%	3	27%	0	0%
High/Moderately high	4	29%	12	100%		1	33%	3	100%	3	27%	9	100%
*Adapt EBI*					< 0.001								
Low/Moderately low	8	29%	0	0%		2	33%	0	0%	6	55%	0	0%
Moderate	3	21%	0	0%		0	0%	0	0%	3	27%	0	0%
High/Moderately high	3	14%	11	100%		1	33%	2	100%	2	18%	9	100%
*Implement EBI*					< 0.001								
Low/Moderately low	7	50%	0	0%		2	33%	0	0%	5	45%	0	0%
Moderate	3	21%	2	17%		0	0%	1	33%	3	27%	1	11%
High/Moderately high	4	29%	10	83%		1	33%	2	67%	3	27%	8	89%

* missing 2 participants

*IB* implementation blueprint

*EBI* evidence-based intervention

### Demographic characteristics of clients

Table 3 shows the demographic characteristics of the clients who participated in the CRC screening EBI workshop delivered by the CBO CHEs. All clients were Hispanic and their mean age was 40 years old. The majority were women (86.4%), born outside of the USA (97.7%), had high school education or less (84.1%), and were uninsured (63.6%). Some had full- or part-time employment (27.3%). Over half of the clients reported an annual household income of less than $30,000. A few clients reported family history of cancer, including breast (2.3%), cervical (4.5%), and CRC (6.8%).

**Table 3:** Demographic characteristics of the clients

Variables (n)	Total (*n* = 44)		CBO 1 (*n* = 22)		CBO 2 (*n* = 22)	
	n	%	n	%	n	%
Age (*n* = 44)						
Age 0–44	11	25.0%	10	66.7%	1	8.0%
Age 45–49	7	15.9%	6	25.0%	1	4.0%
Age 50 +	26	59.1%	6	33.0%	20	92.0%
Gender (*n* = 44)						
Man	5	11.4%	0	0.0%	5	22.7%
Woman	38	86.4%	22	100.0%	16	72.7%
Do not know	1	2.0%	0	0.0%	1	4.0%
Race/ethnicity (*n* = 44)						
White	5	11.4%	2	9.1%	3	13.6%
American Indian/Alaskan Native	1	2.3%	1	4.5%	0	0.0%
Other	26	59.1%	114	63.6%	12	54.5%
I don’t know	7	15.9%	2	9.1%	5	22.7%
Do not want to say	5	11.4%	3	13.6%	2	9.1%
Born in the USA (*n* = 44)						
Yes	1	2.3%	0	0.0%	1	4.5%
No	43	97.7%	22	100.0%	21	95.5%
Mean age immigrated to the USA (*n* = 40)	26.13 (11.82)		27.65 (13.87)		24.6 (9.47)	
Education (*n* = 44)						
None/Kindergarten	5	11.4%	0	0.0%	5	22.7%
1st-8th grade	18	40.9%	13	59.1%	5	22.7%
9–11 grade	8	18.2%	5	22.7%	3	13.6%
12 grade/GED	6	13.6%	1	4.5%	5	22.7%
1–3-yr university	4	9.1%	1	4.5%	3	13.6%
4-yr university	1	2.3%	0	0.0%	1	4.5%
Do not want to say	2	4.5%	2	9.1%	0	0.0%
Way to cover medical costs (*n* = 44)						
No	28	63.6%	15	68.2%	13	59.1%
Yes	14	31.8%	7	31.8%	7	31.8%
Do not want to say	2	4.5%	0	0.0%	2	9.1%
Health insurance (*n* = 14)						
Private	7	50.0%	1	14.3%	6	85.7%
Medicare	1	7.1%	0	0.0%	1	14.3%
Medicaid	1	7.1%	1	14.3%	0	0.0%
Medicare + Medicaid	1	7.1%	1	14.3%	0	0.0%
Other	0	0.0%	0	0.0%	0	0.0%
Do not know	4	28.6%	4	57.1%	0	0.0%
Employment status (*n* = 44)						
Full-time	4	9.1%	0	0.0%	4	18.2%
Part-time	8	18.2%	2	9.1%	6	27.3%
Unemployed	12	27.3%	7	31.8%	5	22.7%
Housewife/Caregiver	11	25.0%	9	40.9%	2	9.1%
Retired	2	4.5%	1	4.5%	1	4.5%
Unable due to health	5	11.4%	2	9.1%	3	13.6%
Do not want to say	2	4.5%	1	4.5%	1	4.5%
Marital status (*n* = 44)						
Married	25	56.8%	10	45.5%	15	68.2%
Divorced	3	6.8%	2	9.1%	1	4.5%
Widower	5	11.4%	3	13.6%	2	9.1%
Separated	1	2.3%	0	0.0%	1	4.5%
Single	2	4.5%	1	4.5%	1	4.5%
Not married, live w partner	6	13.6%	5	22.7%	1	4.5%
Do not want to say	2	4.5%	1	4.5%	1	4.5%
Annual household income (*n* = 44)						
< 10,000	14	31.8%	4	18.2%	10	45.5%
10,000–19,999	3	6.8%	2	9.1%	1	4.5%
20,000–29,999	7	15.9%	4	18.2%	3	13.6%
30,000–49,999	6	13.6%	2	9.1%	4	18.2%
50,000–69,999	5	11.4%	4	18.2%	1	4.5%
Not sure/Don’t know	2	4.5%	1	4.5%	1	4.5%
Do not want to say	7	15.9%	5	22.7%	2	9.1%
Family History of cancer (*n* = 44)						
No	24	54.5%	12	54.50%	12	54.50%
Breast	1	2.3%	1	4.50%	0	0
Cervical	2	4.5%	2	9.10%	0	0
Colorectal	3	6.8%	3	13.60%	0	0
Prostate	0	0.0%	0	0	0	0
Other*	14	31.8%	4	18.20%	10	45.50%
History of inflammatory bowel disease (*n* = 44)						
No	36	81.8%	20	91.0%	16	72.70%
Yes	4	9.1%	0	0	4	18.20%
Unknown	4	9.1%	2	9.10%	2	9.10%

*Other cancers indicated (*n*) = leukemia (1), pancreatic (1), small intestine (1), skin (1), stomach (2), thyroid (2), uterine (1), and type unknown (5)

### Change in CRC and CRC screening knowledge among clients

Table 4 shows significant change in knowledge of CRC and CRC screening among clients pre/post-completion of the CRC EBI workshop. More clients were able to identify correct knowledge about CRC, including slow progression of the disease over time (pre: 72.1% vs post: 93.2%; *p* = 0.01), being the second leading cause of death (pre: 55.6% vs post: 79.5%; *p* < 0.001), not being exclusively hereditary (pre: 31.8% vs post: 63.6%; *p* < 0.001), and reduced risk of colorectal cancer through healthy diet and regular exercise (pre: 81.5% vs. post: 95.5%; *p* = 0.05). There was no significant change pre- and post-evaluation as most participants identified them correctly at pre-evaluation. There was also increased knowledge about CRC screening tests. Post-workshop, clients were more likely to correctly indicate that there is more than one test to detect CRC (pre: 11.4% vs post: 52.3%; *p* < 0.001) and know the recommended age to begin CRC screening (pre: 63.4% vs post: 70.5%; *p* < 0.001). There was no significant difference in clients’ knowledge that screening and early detection improve survival (pre: 90.9% vs post: 97.7%; *p* = 0.26).

**Table 4:** Colorectal cancer screening knowledge

	Total (*n* = 44)				P value	CBO 1 (*n* = 22)				CBO 2 (*n* = 22)			
	Pre		Post			Pre		Post		Pre		Post	
Polyps can turn into cancer over time (n, %)*													
Agree	31	72.1%	40	93.2%	0.01	13	61.9%	19	86.4%	18	81.8%	21	95.5%
Disagree	1	2.3%	1	2.3%		0	0.0%	1	4.5%	1	4.5%	0	0.0%
I am not sure	11	25.6%	3	6.8%		8	38.1%	2	9.1%	3	13.6%	1	4.5%
Colorectal cancer (or colon cancer) is the second leading cause of cancer death in the USA (n, %)**													
Agree	20	55.6%	35	79.5%	< 0.001	7	50.0%	17	77.3%	13	59.1%	18	81.8%
Disagree	1	2.8%	4	9.1%		0	0.0%	3	13.6%	1	4.5%	1	4.5%
I am not sure	15	41.7%	5	11.4%		7	50.0%	2	9.1%	8	36.4%	3	13.6%
Colorectal or warning symptoms appear early in the progress of the disease													
Agree	16	36.4%	24	50.0%	< 0.001	7	31.8%	12	50.0%	9	40.9%	12	50.0%
Disagree	4	9.1%	9	25.0%		1	4.5%	5	25.0%	3	13.6%	4	25.0%
I am not sure	24	54.5%	11	25.0%		14	63.6%	5	25.0%	10	45.5%	6	25.0%
Colorectal cancer (or colon cancer) is exclusively hereditary, there are no risk factors for developing this disease													
Agree	8	18.2%	7	15.9%	< 0.001	2	9.1%	3	13.6%	6	27.3%	4	18.2%
Disagree	14	31.8%	28	63.6%		5	22.7%	15	68.2%	9	40.9%	13	59.1%
I am not sure	22	50.0%	9	20.5%		15	68.2%	4	18.2%	7	31.8%	5	22.7%
A healthy diet and regular exercise can reduce the risk of developing colorectal cancer (n, %)***													
Agree	35	81.4%	42	95.5%	0.05	18	85.7%	22	100.0%	17	77.3%	20	90.9%
Disagree	1	2.3%	0	0.0%		1	4.8%	0	0.0%	0	0.0%	0	0.0%
I am not sure	7	16.3%	2	4.5%		2	9.5%	0	0.0%	5	22.7%	2	9.1%
There is only one test to detect colorectal cancer or colon cancer (n, %)													
Agree	15	34.1%	13	29.5%	< 0.001	8	36.4%	7	31.8%	7	31.8%	6	27.3%
Disagree	5	11.4%	23	52.3%		3	13.6%	14	63.6%	2	9.1%	9	40.9%
I am not sure	24	54.5%	8	18.2%		11	50.0%	1	4.5%	13	59.1%	7	31.8%
A person should begin screening for colorectal cancer (or colon cancer) at age 50 (n, %)													
Agree	28	63.4%	31	70.5%	< 0.001	11	50.0%	15	68.2%	17	77.3%	16	72.7%
Disagree	6	13.6%	12	27.3%		3	13.6%	6	27.3%	3	13.6%	6	27.3%
I am not sure	10	22.7%	1	2.3%		8	36.4%	1	4.5%	2	9.1%	0	0.0%
Survival of colorectal cancer (or colon cancer) can improve with screening and early detection													
Agree	40	90.9%	43	97.7%	0.26	19	86.4%	22	100.0%	21	95.5%	21	95.5%
Disagree	2	4.5%	0	0.0%		1	4.5%	0	0.0%	1	4.5%	0	0.0%
I am not sure	2	4.5%	1	2.3%		2	9.1%	0	0.0%	0	0.0%	1	4.5%

*missing 1 participant pre-test data for CB

**missing 8 participant pre-test data for CBO1

***missing 1 participant pre-test data for CBO1

Clients reported that as a result of participating in the workshop, their knowledge of CRC and CRC screening increased. They also reported that they will likely talk to their doctor about getting screened for CRC and undergo a CRC screening test (Table 5). Clients also reported that they will likely talk with family members and friends about the importance of getting screened for CRC.

**Table 5:** Perception of EBI and CRC screening

	Total (*n* = 44)	CBO 1 (*n* = 22)	CBO 2 (*n* = 22)
Perception of the EBI workshop			
Overall, the workshop was a good experience	1.36(0.49)	1.27 (0.46)	1.47 (0.51)
The presenter knew this topic well	1.52 (0.51)	1.45 (0.51)	1.59 (0.50)
The presenter helped me understand the information	1.48 (0.51)	1.45 (0.51)	1.50 (0.51)
The information presented was just right	2.05 (0.43)	2.09 (0.43)	2.00 (0.44)
EBI on cancer screening			
As a result of participating in the workshop (mean, SD)			
Knowledge about CRC	3.55 (0.70)	3.68 (0.48)	3.41 (0.85)
Knowledge of CRC screening	3.48 (0.70)	3.64 (0.49)	3.32 (0.84)
Likely to be screened for colorectal cancer	3.60 (0.54)	3.67 (0.58)	3.55 (0.51)
Likely to talk to your doctor about getting screened for CRC	3.68 (0.52)	3.73 (0.55)	3.64 (0.49)
Likely to talk with your family about the importance of getting screened for CRC	3.84 (0.37)	3.95 (0.21)	3.73 (0.46)
Likely to talk to your friends about the importance of getting Screened for CRC	3.84 (0.37)	3.91 (0.29)	3.77 (0.43)
Dissemination of EBI			
Recommend this workshop to other people	3.77 (0.42)	3.82 (0.39)	3.73 (0.46)

## Discussion

The Implementation Studio is an implementation support program for rural CHEs that provides facilitation to select, adapt, and implement cancer prevention and control EBIs through didactic training and technical assistance through consultation. This study found that participation in the Implementation Studio increased the knowledge, ability, and confidence of CHEs employed by CBOs to develop and use an implementation blueprint. Additionally, evaluation results support that the rural CHEs were successfully able to select, adapt, and implement a cancer screening EBI after participating in the Implementation Studio. The study also found that clients who received their CBO’s EBI workshop demonstrated greater knowledge about CRC and the benefits of CRC screening, and they reported being more likely to both talk to their doctor about CRC screening and undergo a CRC screening test.

Guided by the literature of capacity building and the Putting Public Health Evidence in Action training, the Implementation Studio was adapted culturally and linguistically to address the capacity of CHEs in rural setting using the cultural and linguistic adaptation framework [13, 14]. Previous studies in capacity building in community settings have focused mostly on urban CBOs [10, 12, 13, 25–28]. Interviews with 70 rural CBOs funded to implement EBI noted their lack of training and technical assistance to support selection of appropriate programs, adaptation to the population they serve, partnerships and buy-in of stakeholders, and alignment of EBIs with the organizations’ mission to ensure sustainability [29]. Our study shows that the implementation studio has potential to increase capacity of CHEs who serve the Hispanic community in rural setting to select, adapt, and implement EBIs. We also found that most CHEs are Spanish language dominant (if bilingual) or Spanish only speakers. Translation of training materials in Spanish language and navigation of online platforms in Spanish language were necessary to address the language needs of the CHEs.

Implementation support strategies, such as the Implementation Studio, focused on facilitating selection of EBIs may reduce the effort needed for the adaptation process from CBO to improve the fit of the EBI to new context. Adaptation encompasses changes made to EBIs to improve their fit or effectiveness to a new population, setting, and context [30]. The CBOs participating in our study received training on how to compare multiple EBIs prior to their selection based on health goals, behaviors, environment, needs, program delivery methods, population of interest, and organizational resources [11]. After EBIs were selected, CBOs made small adaptation that were proactive at pre-implementation. No unplanned or reactive adaptation were made during the implementation as CBOs deemed most content from their selected EBI to be relevant to their community. Implementation researchers have promoted the planned adaptation process as being preferable [31] to avoid unplanned or reactive changes to the EBI as they can affect the fidelity to the intervention [32]. Building the capacity of CBOs to learn to compare multiple EBIs may be an earlier step to the adaptation of EBIs and might help avoid unplanned adaptation, while improving fidelity and fit of the EBI.

More robust EBI repositories are needed in general as well as more specifically for CHEs who are not dominant in the English language. While the EBCCP is an excellent resource for finding EBIs, many EBIs are outdated (e.g., reference out of date screening guidelines), print based, and offer few varieties. Including more language concordant video- or audio-based EBIs can increase the accessibility and appeal of using EBIs among CHEs. Additionally, creating a platform to share and curate EBIs adapted to different populations can help augment the repository, improve the experience of “selecting” the EBI, and increase collaboration across implementation researchers and EBI users [33]. Collaboration with major search providers such as Google™ and Bing™ can potentially lead to better search engines and increased visibility and accessibility of EBIs not included in the repository by the CBOs.

Our study also found that many CHEs were born outside of the USA and spoke Spanish similar to their clients and community members. Online sites that house EBIs such as NCI’s EBCCP need to include an option for navigation in Spanish, a simplified online interface, and a brief video in the landing page that is available in multiple languages, describing the utility of the website, EBIs available, and instructions on how to navigate the website.

This study found that a high number of CHEs and their clients are uninsured. Providing support for building a community–clinic partnership can be a pathway to increase screening rates among CHEs and their clients. While Step 3 of the Studio was focused on building partnerships including clinics, the COVID-19 pandemic made it challenging for CBOs to build partnerships with their local clinics because they were overwhelmed responding to the pandemic. Future Studios will highlight the importance of this step to increase access to and completion of cancer screening tests.

Capacity building training for CHEs can benefit both the CHEs employed by CBOs and their clients. This study conducted individual training for each CBO future research could explore other strategies to promote use and adaptation of EBIs using capacity building strategies including training, technical assistance and group learning [34]. This study focused on CBOs who serve Hispanic immigrants; application of the Implementation Studio to other non-English-speaking communities will shed light into common processes and potentially new processes that require further adaptation for specific communities. Finally, future studies may want to examine whether CHEs who have participated in the Studio are fully equipped to train new CHEs using the train-the trainers’ model.

To our knowledge, this is the first study that delivered capacity building training to implement EBIs in Spanish only or Spanish-dominant CHEs. While Spanish language training is available to increase genetic services for breast and ovarian cancer [35], currently, there is no training on didactics of implementing EBIs with robust technical support during EBI implementation. However, this study has several weaknesses. First, we report a small sample size of CBOs, CHEs, and clients. Future, larger studies are planned to test the Implementation Studio with CBOs in order to expand the sample size and observe whether similar findings hold for other CBOs. Second, we were not able to follow up with the clients to assess whether or not they received a CRC screening test. As the age of some clients who participated in the CBO’s workshop was below the recommended screening age for CRC, ongoing follow-up with this population will inform whether their knowledge gains and CRC screening intentions are sustained and ultimately lead to CRC screening. Similarly, future studies should include long-term follow-up with clients to assess self-reported CRC screening behaviors with clinic verification. Third, the findings of the study did not include post-implementation interviews with CBOs and their clients. A future study is planned to conduct in-depth semi-structured interviews with CBOs and their clients on implementation outcomes of acceptability, barriers to implementation, and sustainability.

## Conclusion

Rural CBOs serving immigrant communities are critical settings for implementing EBIs; rural CBOs are vital partners that can help reduce cancer disparities that are exacerbated by poor social determinants of health that are often experienced by immigrants. The Implementation Studio demonstrated preliminary effectiveness in increasing knowledge, skills, and confidence among rural CBO CHEs in using and implementing EBIs and building their capacity to learn and implement EBIs, respectively. Future delivery of the Studio should address the unique needs of CBOs, including linguistically appropriate materials and resource constraints. Creating a richer repository of language concordant EBIs with online navigation options for non-English-speaking community health workers has the potential to increase accessibility and improve use, experience and sustainability of implementing EBIs.

## Data Availability

Data files and materials pertaining to this publication are available upon request at lindako@uw.edu.

## Abbreviations

CBO: Community-based organization
EBI: Evidence-based intervention
CRC: Colorectal cancer screening
UW: University of Washington
EBCCP: Evidence-based cancer control programs
CHE: Community health educators
PPHEIA: Putting public health evidence in action
